# Antiviral Drug Discovery: Norovirus Proteases and Development of Inhibitors

**DOI:** 10.3390/v11020197

**Published:** 2019-02-25

**Authors:** Kyeong-Ok Chang, Yunjeong Kim, Scott Lovell, Athri D. Rathnayake, William C. Groutas

**Affiliations:** 1 Department of Diagnostic Medicine and Pathobiology, College of Veterinary Medicine, Kansas State University, Manhattan, KS 66506, USA; ykim@vet.ksu.edu; 2 Protein Structure Laboratory, The University of Kansas, Lawrence, KS 66047, USA; swlovell@ku.edu; 3 Department of Chemistry, Wichita State University, Wichita, KS 67260, USA; athridewmina@gmail.com (A.D.R.); bill.groutas@wichita.edu (W.C.G.)

**Keywords:** noroviruses, 3C-like protease, protease inhibitors, antiviral drug development

## Abstract

Proteases are a major enzyme group playing important roles in a wide variety of biological processes in life forms ranging from viruses to mammalians. The aberrant activity of proteases can lead to various diseases; consequently, host proteases have been the focus of intense investigation as potential therapeutic targets. A wide range of viruses encode proteases which play an essential role in viral replication and, therefore, constitute attractive targets for the development of antiviral therapeutics. There are numerous examples of successful drug development targeting cellular and viral proteases, including antivirals against human immunodeficiency virus and hepatitis C virus. Most FDA-approved antiviral agents are peptidomimetics and macrocyclic compounds that interact with the active site of a targeted protease. Norovirus proteases are cysteine proteases that contain a chymotrypsin-like fold in their 3D structures. This review focuses on our group’s efforts related to the development of norovirus protease inhibitors as potential anti-norovirus therapeutics. These protease inhibitors are rationally designed transition-state inhibitors encompassing dipeptidyl, tripeptidyl and macrocyclic compounds. Highly effective inhibitors validated in X-ray co-crystallization, enzyme and cell-based assays, as well as an animal model, were generated by launching an optimization campaign utilizing the initial hit compounds. A prodrug approach was also explored to improve the pharmacokinetics (PK) of the identified inhibitors.

## 1. Introduction

**Figure 1:**
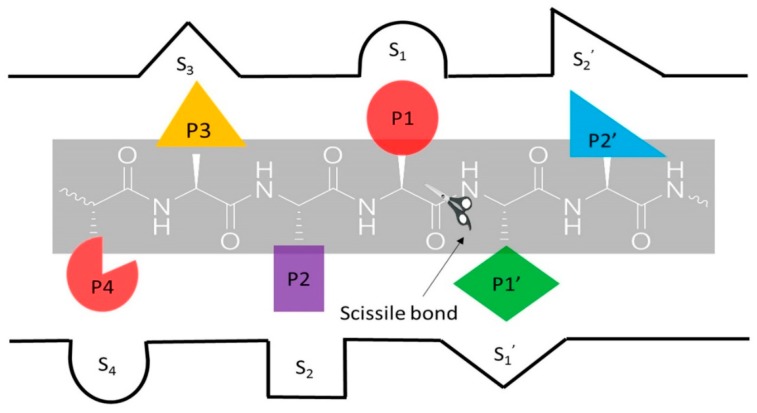
Proteolysis of the scissile bond between P_1_ and P_1_’. Standard nomenclature P_1_… P_n_ and P_1_’… P_n_’ for amino acid residues of the substrates. S_1_... S_n_ and S_1_’… S_n_’ are the corresponding binding sites on the enzyme (per Berger and Schechter nomenclature (3)).

Proteases play an important role in the activation of precursor proteins to mature forms, the recycling of proteins, and other essential functions in the body (vide infra) [1]. They are classified into over 80 families based on evolutionary relationships [1]. The major protease classes based on catalytic types include serine, cysteine, aspartic, threonine, glutamic acid, and metalloproteases [1,2]. Most proteases recognize specific sequences of amino acids in their substrates and cleave the peptide bond between the P_1_ and P_1_’ residues (Figure 1) [3] via nucleophilic attack of the side chain of a cysteine, serine or threonine residue, or a water molecule (aspartic, glutamic acid, and metalloproteases) on the amide carbon of the scissile bond [2].

There are approximately 600 proteases (~2% of the genomes) encoded by the human genome. These are involved in a plethora of important physiological processes, including protein turnover, digestion, blood coagulation, wound healing, fertilization, cell differentiation and growth, cell signaling, the immune response, and apoptosis. However, uncontrolled, poorly regulated, or undesired proteolysis can lead to various diseases [4]. Examples of these include: (1) cancer (metastasis, angiogenesis): proteasome, matrix metalloproteinases (MMPs), caspases, and furin; (2) neurological diseases (Alzheimer’s disease, Parkinson’s disease, ischemia, central nerve system injury): a disintegrin and metalloproteinase domain-containing protein 10 (ADAM10), γ-secretase, β-secretase 1, and MMP-24; (3) inflammation (arthritis, chronic obstructive pulmonary disease (COPD), asthma, Crohn’s disease, inflammatory bowel disease, colitis, diverticulitis, chronic liver disease): MMPs, cathepsins, neutrophil elastase, and ADAM17; (4) blood coagulation: thrombin, factor Xa; (5) diabetes: dipeptidyl peptidase 4 (DPP4); (6) cardiovascular disease (atherosclerosis, hypertension, cardiomyopathy, congestive heart failure, myocardial infarction, neovascularization and cardiac remodeling, cardiac fibrosis, aneurism, ischemia-reperfusion injury): angiotensin converting enzyme, renin, MMPs, chymase, neprilysin, calpain; (7) osteoporosis: cathepsin K; and (8) autoimmune diseases (multiple sclerosis, lupus, Guillain-Barre syndrome, psoriasis): activated protein C, kallikreins; ageing (skin), MMPs, neutrophil elastase [1,2].

**Figure 2:**
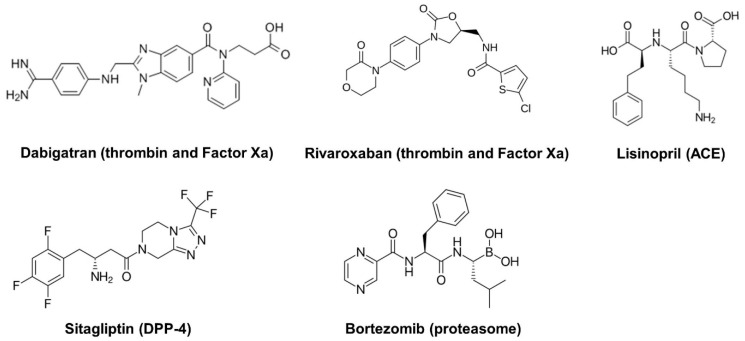
Examples of commercially available protease inhibitors and their targets. Dabigatran and Rivaroxaban are anti-coagulants; Lisinopril is for hypertension; Sitagliptin is for diabetes; and Bortezomib is for multiple myeloma.

Since poorly-regulated proteases themselves can be detrimental, there are physiological protease inhibitors in the body which help to maintain a protease–protease inhibitor balance by controlling aberrant protease activity [5,6,7]. Well-known physiological protease inhibitors include the serpin superfamily, consisting of α-1-antitrypsin, α-1-antichymotrypsin, C1-inhibitor, antithrombin, plasminogen activator inhibitor-1, and neuroserpin [6,7]. Thus, natural or synthetic protease inhibitors have been studied and developed for various pathological conditions [4] and there are several FDA-approved drugs on the market. Currently, commercially available synthetic protease inhibitors targeting host proteases include (1) thrombin and Factor Xa inhibitors for coagulation (such as dabigatran and rivaroxaban); (2) angiotensin converting enzyme (ACE) inhibitors for hypertension (such as lisinopril); (3) DPP-4 inhibitors for diabetes (such as sitagliptin); and (4) proteasome inhibitors for multiple myeloma (such as bortezomib) (Figure 2).

## 2. Human Immunodeficiency Virus (HIV) Protease Inhibitors

**Figure 3:**
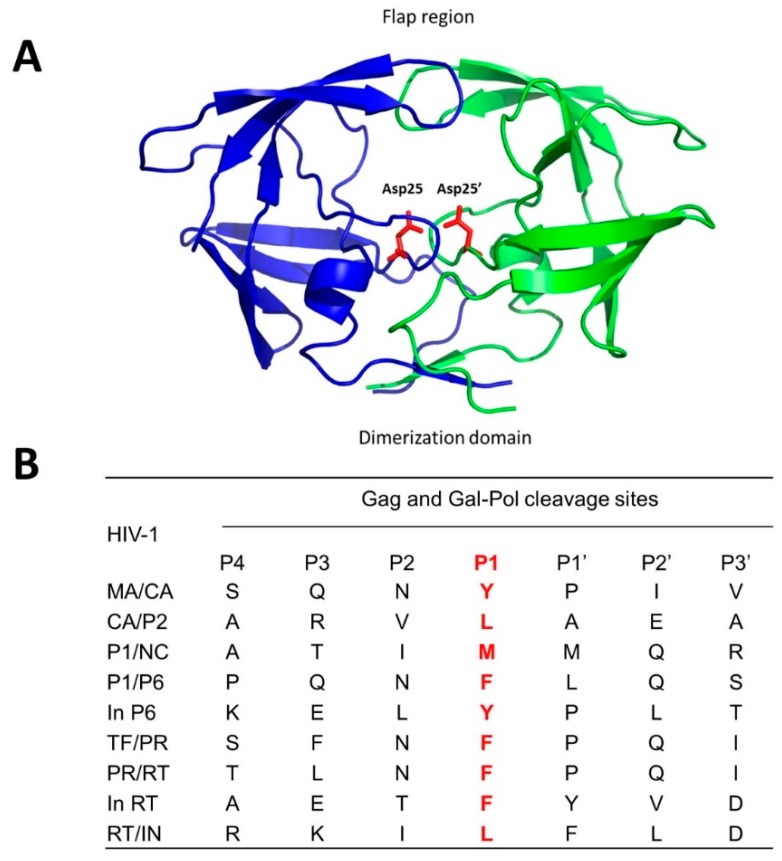
Crystal structure of HIV-1 protease (**A**), and its cleavage sites (**B**). The catalytic site resides between two monomers (blue and green), and Asp25 and Asp25’ (red) from each dimer orchestrate the cleavages (PDB: 2NMZ). The flap region and dimerization domain are also shown. Cleavage sites (amino acids) of mature proteins of HIV-1 are listed. The red color indicates P_1_ specificity.

Certain viruses also encode viral proteases for successful infection of the hosts. The HIV aspartic and hepatitis C virus (HCV) NS3-4A serine protease are among the well-studied due to their significance in public health and successful antiviral drug development. Each HIV particle has two copies of positive-sense, single-stranded RNA genome. Upon infection of host cells, the HIV-1 genome is transcribed into double-stranded DNA through reverse transcription, which is then integrated into the human genome. The HIV-1 virus utilizes the host cell machinery to replicate, producing virus particles. As viruses are released from host cells, they undergo maturation where viral structural polyproteins are cleaved by HIV-1 viral protease to produce infectious virus particles. The functional HIV protease is formed by two identical monomers of 99 amino acids. Each protease monomer contributes one catalytic aspartic acid (25 and 25’ Asp) in the active site (Figure 3A), and the two conserved catalytic residues work together to process HIV-1 Gag and Gag-Pol polyproteins during viral maturation when new virus particles are released from cell membrane. The active site is covered by two flexible β-hairpin flaps (Figure 3A), which open to allow the substrates to enter the active site [8]. HIV-1 protease cleaves the Gag and Gag-Pol polyprotein precursors at nine processing sites to produce mature proteins (Figure 3B) [8]. In general, a hydrophobic amino acid at P_1_ is required for efficient cleavage by HIV-1 protease, and the cleavage rates vary significantly based on the sequences in the P_6_ to P_3_’ region [9,10].

**Figure 4:**
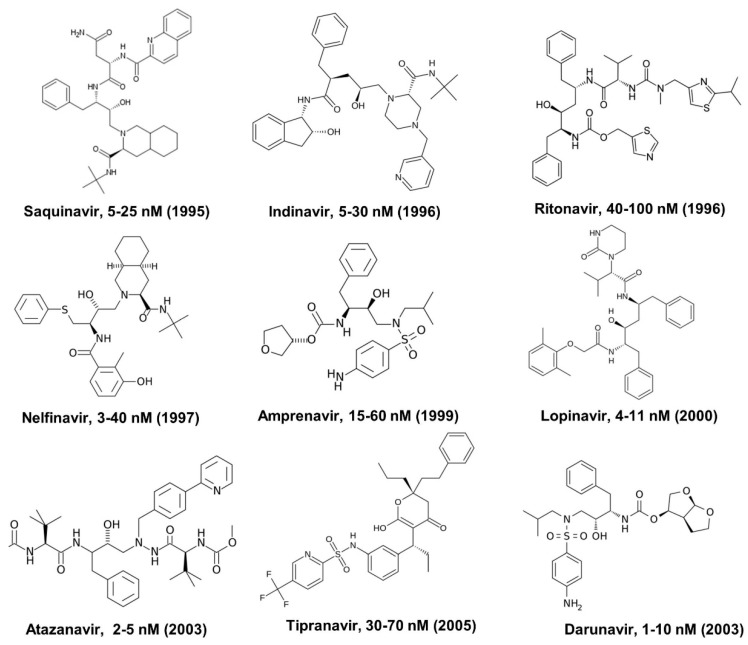
FDA-approved HIV protease inhibitors with approved year and EC_50_ values against HIV-1 in cell culture.

Since maturation of HIV-1 by viral protease is essential to produce infectious virus particles, HIV-1 protease has been the target of extensive drug discovery and development efforts. There are at least ten HIV protease inhibitors approved by the FDA, including saquinavir, indinavir, ritonavir, nelfinavir, amprenavir, fosamprenavir, lopinavir, atazanavir, tipranavir, and darunavir (Figure 4) [11,12]. These compounds are peptidomimetic inhibitors that target the active site, except for Tipranavir which binds to the flap region of the viral protease [11]. The strategies employed in targeting the viral protease active site utilize reduced small peptides (compounds with 3 to 5 peptidyl residues containing a hydrophobic residue at the P_1_ position) with an isosteric replacement at the scissile bond which mimics the tetrahedral transition state of the proteolytic reaction [8]. Most commercially available drugs use a nonhydrolyzable hydroxyethylene or hydroxyethylamine moiety as the basic core [8] (Figure 4). Other examples of noncleavable transition-state isosteres are statine, norstatine, phosphinate, reduced amide, dihydroxyethylene, α-keto amide and, more recently, silicon-based inhibitors [8]. The 50% effective concentration (EC_50_) values of these commercially available inhibitors against HIV-1 in cell culture range from 1 to 100 nM (Figure 4). To enhance the physicochemical and PK properties of protease inhibitors, a prodrug approach has also been utilized. For example, Fosamprenavir is the phosphate ester prodrug of amprenavir that contains a hydrolyzable ester bond. The phosphate prodrug displays improved cellular absorption and diminished side effects [13]. Additionally, protease inhibitors are used in combination antiretroviral therapy (cART) regimes.

## 3. Hepatitis C Virus (HCV) Protease Inhibitors

**Figure 5:**
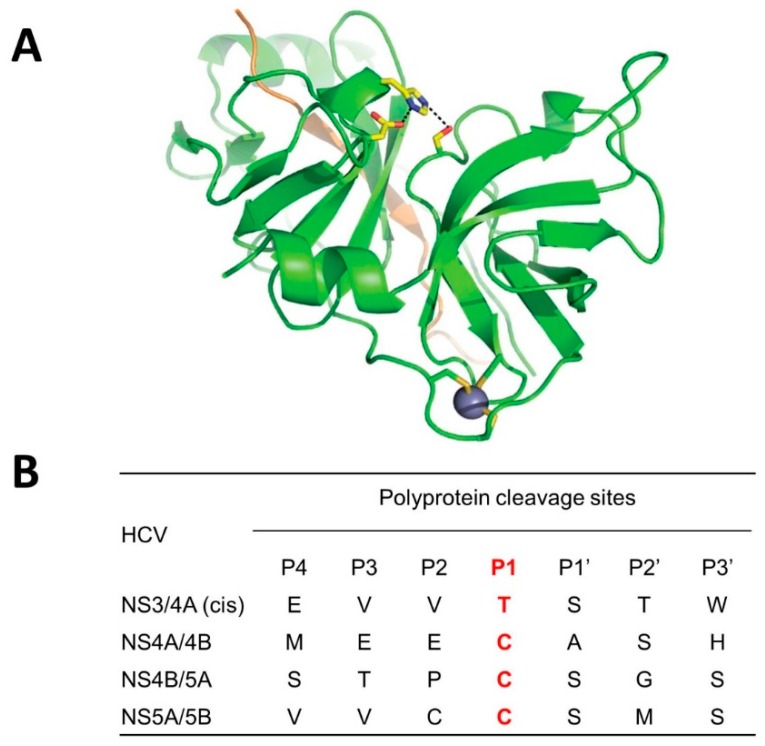
Crystal structure of hepatitis C virus (HCV) NS3/4A (genotype 1), and its cleavage sites. (**A**) The two β-barrel sub-domains of the NS3/4A protease domain are indicated in a ribbon diagram (adapted from PDB:1A1R). His57, Asp99, and Ser139, which form the catalytic triad, are shown in ball-and-stick representation. The protease structural zinc ion (blue sphere) is indicated. The NS4A peptide is rendered as a light orange ribbon. (**B**) Cleavage sites (amino acids) between mature proteins of HCV genotype 1 are listed. Red color indicates P_1_ specificity.

HCV is a single stranded, positive-sense RNA virus, and its genome is approximately 9.6 kb, which is translated into a polyprotein of ~3000 amino acid residues. There are at least six distinct HCV genotypes (1 to 6) with genotype 1 accounting approximately 70 to 75% of all HCV infections in the US [14]. The polyprotein is processed by cellular and viral proteases to generate at least 10 mature structural and non-structural proteins. HCV encodes an NS2-3 autoprotease and a NS3-4A serine protease. NS2-3 protease autocleaves the NS2 and NS3 junction and NS3-4A serine protease is responsible for cleavage at four junctions on viral polyproteins (NS3-NS4A, NS4A-NS4B, NS4B-NS5A, and NS5A-NS5B junctions), as well as cellular proteins that are involved in innate immunity. NS3 is comprised of protease (N-terminal, ~200 aa) and helicase domains (C-terminal), and forms a heterodimer with NS4A (54-residue protein) [15,16]. NS4A binds to the N-terminal region of NS3 and acts as a cofactor of the protease to enhance cleavage [15,16]. NS3 forms a trypsin-like fold with two β-barrels and contains the catalytic triad residues (His57, Asp81 and Ser139) in the active site (Figure 5). The cleavage sites by NS3-4A protease on the polyprotein are listed in Figure 5 [17]. The P_1_ residue is accommodated in the S_1_ pocket in the NS3-4A protease, and other residues flanking the pocket are important determinants of substrate recognition. The S_1_ pocket of NS3 protease prefers small, hydrophobic residues (such as cysteine) at the P_1_ position of its substrates [17]. HCV NS3-4A is known to have a shallow binding pocket requiring an extended subtrate spanning 10 amino acid residues (P_6_ to P_4_’) for efficient enzyme activity. The consensus sequence for P_6_ to P_1_’ is (D/E)XXXX(C/T)(A/S) for all trans-cleavage sites, with X being any amino acid.

**Figure 6:**
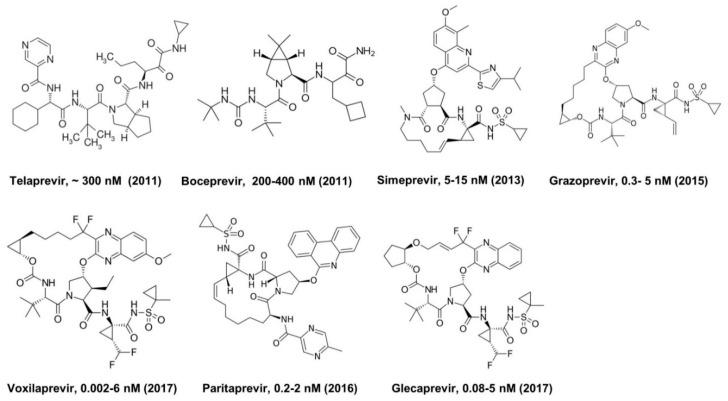
FDA-approved HCV protease inhibitors with approved year and EC_50_ values against genotype 1 in cell culture (replicon harboring cells).

Commercially available HCV protease inhibitors include Telaprevir (Incivek) [18], Boceprevir (Victrelis) [19], Simeprevir, Grazoprevir, Voxilaprevir, Paritaprevir, and Glecaprevir [20] (Figure 6). These inhibitors are often used as part of combination therapy with other inhibitors, such as polymerase and NS5A inhibitors. The macrocyclic compounds, shown in Figure 6, display significantly improved EC_50_ values over Telaprevir and Boceprevir, the first-generation protease inhibitors [20] (Figure 6). Both Boceptrevir and Telatrevir contain an α-ketoamide warhead, a small residue at the P_1_ position, and a Pro or Pro surrogate at the P_2_ position [18,19] (Figure 6). Structure-activity relationship (SAR) studies with HCV protease inhibitors have demonstrated a preference for residues with small hydrophobic side chains such as ethyl, propyl and trifluoroethyl at the P_1_ position and bulky substituents at the P_2_ position for increased efficency. SAR studies utilizing different warhead groups in protease inhibitors have shown that an α-ketoamide warhead is preferred, resulting in up to a 40-fold improvement in binding affinity over the aldehyde counterpart [21]. Other warheads, such as trifluoromethyl or chloromethyl ketones, were found to be less effective [20]. The EC_50_ values of these protease inhibitors ranged from 100 to 300 nM for Telatrevir and Boceprevir, and from 0.2 to 15 nM for the macrocyclic compounds in HCV replicon harboring cells, dependent on the virus genotypes used (Figure 6).

## 4. Norovirus 3CL Protease (NV 3CL^Pro^) Inhibitors

Human noroviruses causing acute gastroenteritis belong to two major genogroups (GI and GII) comprised of at least 28 genotypes associated with human disease [22]. As a GI strain, Norwalk virus (NV) is the first known norovirus, and serves as a prototype strain [23]. Currently, the GII genogroup viruses are more prevalent, and GII.4 and newly emerging GII.17 strains are primarily responsible for most infections and outbreaks of acute gastroenteritis [24,25]. Outbreaks occur frequently in hospitals, nursing homes, navy and cruise ships, and schools, and they are difficult to control due to the highly contagious and genetically diverse nature of noroviruses, as well as their prolonged shedding and high stability in the environment [3,26]. The most common routes of virus transmission are fecal–oral, food- or waterborne, aerosol and person-to-person [23,27]. The vomiting and diarrhea associated with norovirus infection is incapacitating but self-limiting among healthy adults. However, morbidity is high in immunocompromised individuals, young children, and the elderly [28,29]. The significant impact of noroviruses on public health and potential bioterrorism threat underscores the need for norovirus-specific small molecule therapeutics and prophylactics.

**Figure 7:**
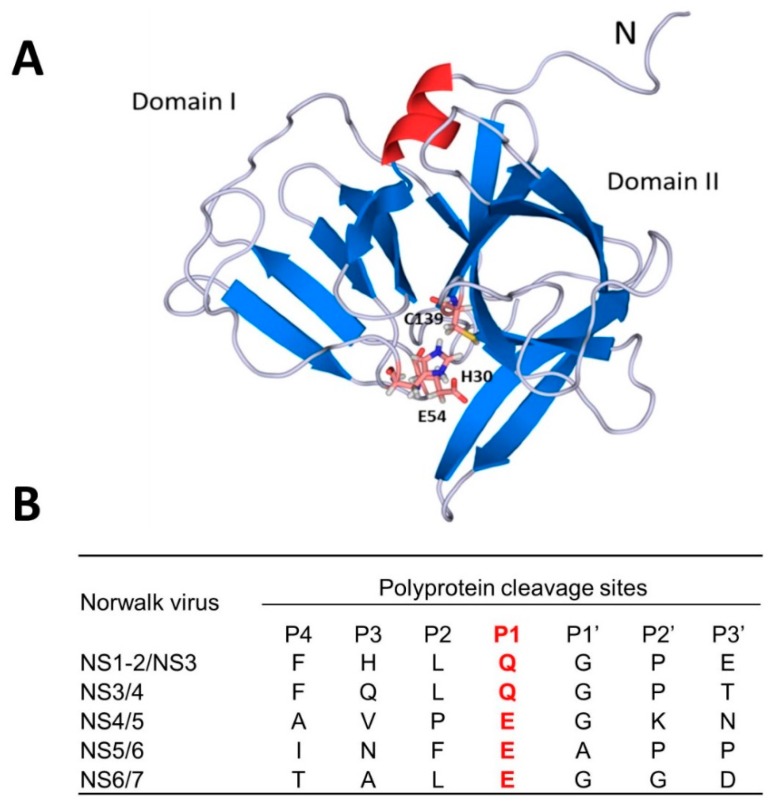
Crystal structure of NV 3CL^Pro^ (PDB: 2LNC) (**A**) and its cleavage sites (**B**). The active site resides between the two domains and the catalytic triad with Cys139, His30 and Glu54 are shown. Cleavage sites (amino acids) between mature proteins of Norwalk virus are listed. The red color indicates P_1_ specificity.

The genome of noroviruses contains a single-stranded, positive-sense RNA that consists of three open reading frames (ORFs) that encode a 200 kDa polyprotein (ORF1), a major capsid protein VP1 (ORF2), and a small basic protein VP2 (ORF3) [23]. The polyprotein is processed by a virus-encoded 3C-like cysteine protease (3CL^Pro^) to generate mature non-structural proteins. Norovirus 3CL^Pro^ forms a typical chymotrypsin-like fold and contains the catalytic residues of Cys139, His30 and Glu54 in the active site (Figure 7A). Noroviruses are classified into seven genogroups (GI–GVII) [23,25]. However, the active sites of norovirus 3CL^Pro^ from diverse noroviruses and related caliciviruses, as well as coronaviruses (3C-like proteases) and picornaviruses (3C proteases), are well-conserved [30]. The mechanism of action of norovirus 3CL^Pro^ is similar to that established for related cysteine proteases where Cys139 acts as a nucleophile, His30 functions as a general acid/base, and Glu54 facilitates the alignment of His30 and promotes deprotonation of Cys139. The oxyanion of the tetrahedral intermediate is stabilized by the presence of an oxyanion hole adjacent to the norovirus 3CL^Pro^ active site. The substrate specificity of norovirus 3CL^Pro^ shows a strong preference for a D/E-F-X-L-Q-G-P sequence (where X is H, Q or V), corresponding to the subsites S_5_-S_4_-S_3_-S_2_-S_1_-S_1_’-S_2_’ (Figure 7B).

### 4.1. Structure-Guided Optimization of Dipeptidyl Inhibitor Lead Series

**Figure 8:**
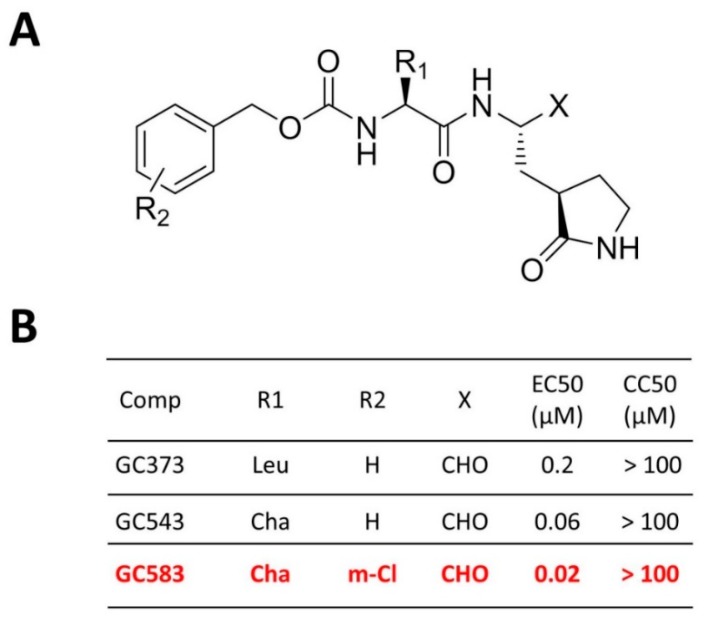
Examples of optimization strategy employed for the dipeptidyl compounds against norovirus from one of the initial hits (*GC373*). Potency increased by making changes in R_1_ and R_2_. Red color indicates the most optimized *GC583*.

The general strategy employed in the design of norovirus 3CL^Pro^ inhibitors involved the construction of transition-state (TS) inhibitors or TS mimics (Figure 8). We have synthesized dipeptidyl and tripeptidyl compounds containing a glutamine surrogate at the P_1_ position with numerous variations at the P_2_ and/or P_3_ positions. The efficacy of each inhibitor was evaluated using norovirus 3CL^Pro^ from GI (Norwalk virus, NV), GII (MD145) and GV (murine norovirus-1, MNV-1), as well as cell-based assays with NV replicon harboring cells [31] and MNV-1 [32]. The antiviral effects of each inhibitor were also evaluated using feline calicivirus in cell culture to assess their ability to inhibit diverse noroviruses and caliciviruses [30]. The inclusion of a glutamine surrogate at the P_1_ position was found by us, and others, to be crucial for efficient inhibitory activity. An array of warheads (the reactive residue that interacts with the catalytic Cys139) was employed in the synthesis of inhibitors, including aldehyde, α-ketoamide, α-ketoester, nitrile, and α-ketoheterocycle moieties [33,34]. Moreover, TS mimics such as α-hydroxyphosphonates and α-hydroxyesters, were also investigated [35]. The synthesized inhibitors were examined for inhibitory activity in the fluorescence resonance energy transfer (FRET) protease assay using norovirus 3CL^Pro^ from GI and GII strains [36], NV replicon harboring cells [31], and against MNV-1 in RAW267.4 cells [32]. These studies showed that although most of these warheads were active against norovirus 3CL^Pro^, the aldehyde warhead displayed superior activity (>3–5 fold) compared to other warheads. The high chemical reactivity of aldehyde warheads employed in the design of TS inhibitors is occasionally associated with a lack of selectivity, as well as off-target effects. Therefore, a latent aldehyde warhead, such as an aldehyde bisulfite adduct, which reverts to the precursor aldehyde under physiological conditions was utilized [37]. The pharmacological activity of the bisulfite adducts of peptidyl aldehydes was shown to be comparable to those of the precursor aldehydes [37].

### 4.2. Optimization at the P2 and P3 Positions

**Figure 9:**
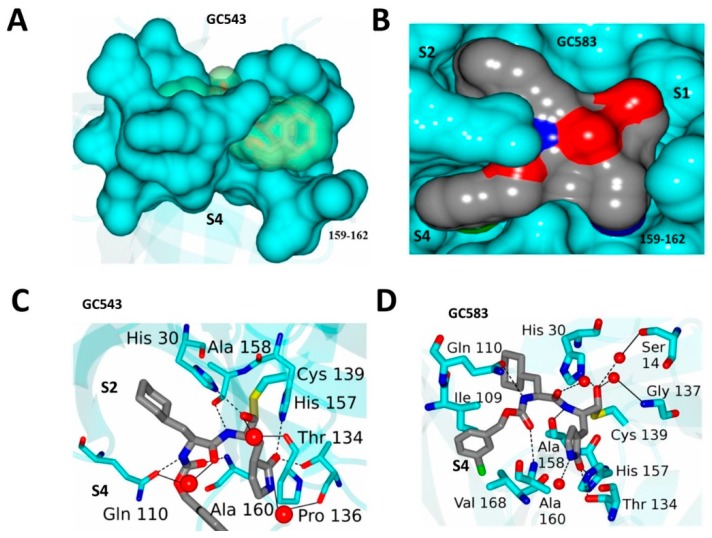
X-ray crystal structure of NV 3CL^Pro^ and *GC543* (**A**,**C**, PDB: 4XBC) and *GC583* (**B**,**D**, PDB: 4XBB). The structures revealed that increased potency is correlated to interactions between the S_4_ subsite and the cap residue. The *m*-Cl benzyl group in the cap allows for interactions with hydrophobic residues in S_4_.

Crystal structures of NV 3CL^Pro^ in complex with dipeptidyl inhibitors (including *GC373*) revealed that a large conformational change occurs in a nearby active site loop to accommodate inhibitor binding [30]. These structures served as a guide for further development of potent dipeptidyl inhibitors. Using *GC373* as a starting point [33], optimization of R_1_ (accommodated in the S_2_ pocket) and R_2_ (accommodated in the S4 pocket) was conducted (Figure 8) [38]. The FRET assay using 3CL^Pro^ of GI and GII noroviruses (IC_50_) and cell based assays (EC_50_) using NV replicon harboring cells revealed that replacing Leu at R_2_ with cyclohexylalanine (Cha) (*GC543*) increased potency significantly (IC_50_ 600 nM to 300 nM and EC_50_ 200 nM to 60 nM) (Figure 8) [38], while the substitution of R_2_ with a *m*-Cl substituent further increased potency (IC_50_ 100 nM and EC_50_ 20 nM) (*GC583*) [38]. Co-crystal structures with each compound revealed the structural determinants that are responsible for the increase in potency (Figure 9): (1) a more extensive hydrogen bonding network is present relative to *GC373*; (2) the cyclohexyl ring is positioned tightly, optimally filling the hydrophobic S_2_ subsite of NV Pro; and (3) the *m*-chlorophenyl ring occupies a hydrophobic pocket near Ile109 and Val168 [38]. The EC_50_ values against MNV-1 were also found to be well correlated with the EC_50_ values in NV replicon harboring cells (HG23 cells): 3.5 µM, 0.6 µM, 0.08 µM for *GC373*, *GC543* and *GC583*, respectively [38]. Additionally, SAR studies were performed to probe the hydrophobic S_4_ pocket of norovirus 3CL^Pro^ and exploit favorable hydrophobic binding interactions [39]. Specifically, because the *m*-Cl benzyl moiety in *GC583* projects toward the S_4_ subsite of the protease (Figure 9), its close proximity to a string of hydrophobic amino acids (Ala158, Ala160, Val168 and Ile109) was exploited through appropriate cap modifications, including the use of sulfonamide and lipid moieties [39]. The synthesized compounds displayed high potency in inhibiting norovirus replication in cells (EC_50_ up to 0.1 µM in replication in NV harboring cells or MNV-1) but did not increase the potency over *GC583* [39].

### 4.3. Prodrug Approach

The prodrug approach has been highly successful in improving the absorption, distribution, metabolism, and excretion (ADME)/PK characteristics of HIV and HCV protease inhibitors. Our group utilized aldehyde bisulfite adduct inhibitors to synthesize ester and carbamate prodrugs to improve their PK and pharmacodynamic (PD) properties [26]. The generated prodrugs were found to have comparable efficacy to their precursors, and exhibited lower or low cytotoxicity, increased stability in liver microsomes and reduced plasma protein binding, demonstrating the soundness of the prodrug approach in the development of NV 3CL^Pro^ therapeutics, as well as its general applicability to other viral serine and cysteine proteases of medical relevance [26]. For the dipeptidyl inhibitors against norovirus 3CL^pro^, the hydroxyl group in bisulfite adducts provided a convenient site for further derivatization to yield ester and carbamate prodrugs [26]. Enzyme-mediated or nonenzymatic hydrolysis converts prodrugs into the aldehyde bisulfite adduct, which subsequently reverts to the precursor aldehyde [26].

### 4.4. Macrocyclic Inhibitors Targeting Norovirus 3CL^Pro^

**Figure 10:**
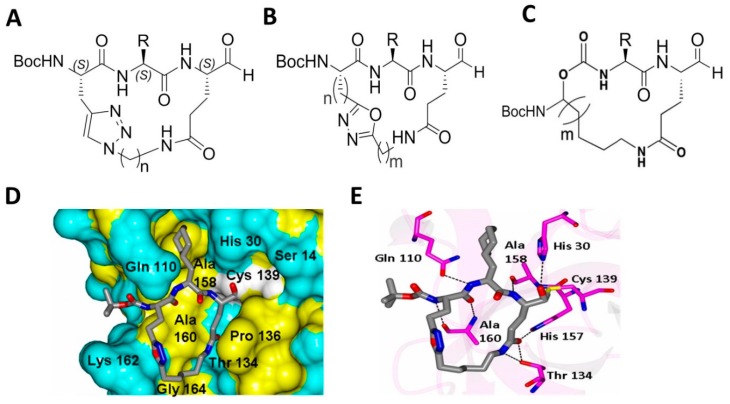
Examples of macrocyclic NV 3CL^Pro^ inhibitors. (**A**–**C**). Three different classes (**A**–**C**) of macrocyclic inhibitors were synthesized. The P_2_ position, and the length and nature of the linker were examined for optimal efficacy against the enzyme and viral replication. (**D**–**E**) X-ray co-crystallization of NV 3CL^Pro^ and an oxadiazole-based macrocycle (**B**, PDB: 5DG6).

Macrocyclic compounds frequently display good pharmacological activity and selectivity, enhanced permeability, and improved metabolic stability; consequently, there has been an intense interest in macrocyclic inhibitors and their utilization in drug discovery and development. The design rationale of macrocyclic norovirus 3CL^Pro^ inhibitors entailed the following considerations: firstly, proteases are known to recognize their ligands in the β-strand conformation [40]; therefore, macrocyclization is an effective way of pre-organizing a peptidyl transition-state mimic in a β-strand conformation suitable for binding to the active site of a protease [41]. This invariably increases affinity by reducing the loss of entropy upon inhibitor binding. Secondly, macrocyclization increases cellular permeability, particularly when the design includes the formation of an intramolecular hydrogen bond, as well as proteolytic stability [42]. Thus, macrocyclization generally enhances drug-like characteristics [43]. For norovirus 3CL^Pro^, the plasticity of the S_3_ subsite allows the design of macrocyclic inhibitors by tethering the P_1_ Gln side chain to the P_3_ residue side chain. Based on these considerations, our group synthesized and evaluated for the first time a series of novel classes of macrocyclic compounds for their antiviral effects against noroviruses (Figure 10) [44,45,46,47]. These include (1) triazole-based macrocyclic inhibitors [45]; (2) oxadiazole-based macrocyclic inhibitors [47]; and (3) macrocyclic inhibitors incorporating a methylene linker [46] (Figure 10). These non-optimized macrocyclic compounds were found to be cell permeable and displayed moderate inhibitory activity against NV 3CL^Pro^ and viral replication in cells [44,45,46,47]. Their EC_50_ ranged between 2 to 100 µM in NV replicon harboring cells [44,45,46,47]. X-ray co-crystallization with the macrocyclic compounds showed that, in some of the compounds, the number of hydrogen bonds between NV 3CL^Pro^ and the inhibitors decreased in comparison with the non-macrocyclic dipeptidyl compounds arising from a major structural reorganization of the active site (Figure 10). Pharmacological activity and cellular permeability were found to be dependent on the interplay of several factors, including the ring size, type of linker, and other structural determinants.

### 4.5. Tripeptidyl Inhibitors

Our group reported for the first time that Rupintrivir, an enterovirus 3C protease inhibitor, was also effective against norovirus replication [30]. Rupintrivir is a tripeptidyl compound possessing an α, β-unsaturated ester (a Michael acceptor) as a warhead which inactivates norovirus 3CL^Pro^ irreversibly, presumably through a Michael addition reaction with Cys139 [48]. Several tripeptidyl compounds were synthesized with a glutamine surrogate at the P_1_ position and their effects on norovirus in the fluorescence resonance energy transfer (FRET) and cell-based assays were examined [49]. SAR studies with the tripeptidyl series of compounds showed that, like dipeptidyl inhibitors, a cyclohexyl alanine at the P_2_ position increased potency [49]. In addition, the cellular permeability of the tripeptidyl compounds was dependent on the nature of the residue at the P_3_ position. Compounds with a hydrophobic residue at P_3_ (such as naphthyl alanine and Phe) were highly effective in the FRET and cell-based assays (the best compound was *NPI52* with an EC_50_ of 0.04 µM in the replicon harboring cells). Detailed structures and the efficacy of the tripeptidyl compound series are reported in our prior report [49]. Similar tripeptidyl compounds with acyclic amides [50] or a 6-membered lactam ring [51] at the P_1_ position were synthesized and evaluated for their anti-norovirus effects. However, their efficacy was lower than that of *NPI52* in enzyme- or cell-based assays [49,50,51].

### 4.6. Potential of Dipeptidyl Compounds as Antiviral Drugs

Feline infectious peritonitis (FIP) is caused by a virulent feline coronavirus and is highly fatal (100% fatality). In cats with FIP, granulomatous vasculitis and granuloma lesions composed mainly of virus-infected macrophages are found in various organs, leading to clinical signs, which may include characteristic bodily effusions. The absolute lymphopenia, a prominent feature of both experimental and natural infection of FIP, is associated with the massive apoptosis of uninfected T-cells and its appearance precedes clinical signs typical of FIP. Due to the conservation of 3C proteases from picornaviruses, and 3CL^pro^ from caliciviruses and picornaviruses, most dipeptidyl and tripeptidyl compound series were also effective against multiple viruses in these families [30]. Since *GC376* (bisulfite adduct of corresponding aldehyde *GC373*) potently inhibits feline infectious peritonitis virus (FIPV) in cell culture (EC_50_ 0.04 µM with FIPV 1146 strain) [52], we determined the efficacy of *GC376* against FIP in cats as a proof-of-concept study using experimentally-infected pathogen-free (SPF) cats and client-owned cats with natural infection with FIPV [53,54]. These studies have demonstrated that (1) *GC376* was well tolerated in the animals with up to 4-week continual treatments and (2) for the first time, drug-like small-molecule inhibitors (*GC376*-like molecules) of coronaviruses and noroviruses can serve as potential antiviral therapeutics.

## 5. Conclusions

Proteases are proven therapeutic targets for antivirals. Our group has been working on the development of protease inhibitors against noroviruses for the past several years. These are rationally designed transition-state inhibitors consisting of dipeptidyl, tripeptidyl and macrocyclic compounds. These highly effective inhibitors, validated by X-ray co-crystallization, enzyme and cell-based assays, as well as an animal model, were generated by an optimization campaign utilizing the initial hit compounds. These findings warrant further development of the cited series of compounds beyond preclinical testing.
